# Understanding Ebola: the 2014 epidemic

**DOI:** 10.1186/s12992-016-0194-4

**Published:** 2016-09-13

**Authors:** Jolie Kaner, Sarah Schaack

**Affiliations:** 1 Department of Epidemiology, Oregon Health and Science University, 3181 S.W. Sam Jackson Park Rd., Portland, OR 97239 USA; 2 Department of Biology, Reed College, 3203 SE Woodstock Blvd, Portland, OR 97202 USA

**Keywords:** Ebola, *Ebolavirus*, 2014 outbreak, Epidemic, Review

## Abstract

Near the end of 2013, an outbreak of *Zaire ebolavirus* (EBOV) began in Guinea, subsequently spreading to neighboring Liberia and Sierra Leone. As this epidemic grew, important public health questions emerged about how and why this outbreak was so different from previous episodes. This review provides a synthetic synopsis of the 2014–15 outbreak, with the aim of understanding its unprecedented spread. We present a summary of the history of previous epidemics, describe the structure and genetics of the *ebolavirus*, and review our current understanding of viral vectors and the latest treatment practices. We conclude with an analysis of the public health challenges epidemic responders faced and some of the lessons that could be applied to future outbreaks of Ebola or other viruses.

## Background

As of April 13th, 2016 there have been 28,652 total cases of Ebola virus disease (EVD; or more generally Ebola) in the 2014–2015 West African epidemic [1]. Of these, 11,325 cases (40 %) were fatal [1]. During this epidemic, the vast majority of cases were concentrated in Guinea, Liberia, and Sierra Leone, with a handful of cases imported to countries around the world [1]. This was the first outbreak of Ebola in West Africa, and the most significant Ebola epidemic that has occurred worldwide since the virus was first described [2]. Here, we review the current understanding of biology and genetics of the virus, the past and current epidemiology, and the public health response to the 2014–15 Ebola outbreak.

### *Ebolavirus* genetics

The *ebolavirus* is a member of the family filoviridae, which is composed of single-stranded negative-sense enveloped RNA viruses [3]. These filamentous viruses are ~19 kilobases (kb) in length (800–1100 nanometers [nm] long and 80 nm in diameter) [4]. The *ebolavirus* genome contains seven genes (3′ NP VP35 VP40 GP VP30 VP24 L 5′ [5]) encoding a number of proteins: *NP* (nucleoprotein), *VP35* (polymerase cofactor), *VP40* (matrix protein), *GP* (glycoprotein), *VP30* (transcription activator), *VP24* (secondary matrix protein), and RNA-dependent RNA polymerase [6]. There are currently five recognized species of *ebolavirus*: *Zaire ebolavirus* (ZEBOV), *Sudan ebolavirus* (SEBOV), *Reston ebolavirus* (REBOV) (non-pathogenic to humans), *Côte d’Ivoire ebolavirus* (CIEBOV) also known as *Tai Forest ebolavirus* and *Bundibugyo ebolavirus* (BEBOV) [5, 7].

Estimates of the rate of nucleotide substitution for *filoviruses* suggest that these viruses have substitution rates approximately 100× times lower than other RNA viruses (e. g. retroviruses and influenza A) [8]. Based on these substitution rates, studies have concluded that *ebolavirus* and *marburgvirus*, a closely related *filovirus* that is also pathogenic in humans, likely diverged from each other several thousand years ago and that the different species of *ebolavirus* diverged from each other within the last ~1000 years ago [8, 9]. Genetic analysis of strains from the 2014–2015 West African Ebola epidemic have been hindered, in part, due to the limited understanding of the biology of this virus and further exacerbated by delays in sample export, bad record keeping, and a small number of trained specialists [10, 11]. Gire et al. [12] analyzed 81 EBOV sequences, 78 newly derived from patients in Sierra Leone and 3 previously published Guinean sequences, and found 341 fixed substitutions and 55 single-nucleotide polymorphisms. They concluded that the substitution rate during this outbreak is roughly twice as high as those previously reported. Other studies show contrasting results, however; e.g. Spielman et al. [13] and Hoenen et al. [14] analyzed sequences from the recent epidemic and reported a mutation rates of 9.6 × 10^−4^ and 1.3 × 10^−3^ substitutions per site per year, respectively, which are similar to those reported during past outbreaks.

A more recent study by Carroll et al. [15] used 197 new viral sequences in addition to publicly-available sequences to trace the evolution of the viral genome throughout the epidemic. They estimated the date of the most recent common ancestor of the sampled viruses to be between December 12^th^, 2013 and February 18^th^, 2014, which is supported by epidemiological evidence that places the index case in late December 2013 [15]. Another recent study by Simon-Loriere et al. [16] analyzed 85 new sequences from Guinean patients along with 110 publicly-available EBOV sequences from this outbreak and reported a mutation rate of 0.87 × 10^−3^ to 0.91 × 10^−3^ substitutions per site per year. They point out that evolutionary rates in RNA viruses can be strongly time-dependent, with higher rates observed over short time spans than long ones [16]. This may explain why certain estimates of mutation rates during this recent epidemic have been higher than expected.

### Past Ebola outbreaks

Since the first outbreak 40 years ago, EVD outbreaks have been rare, small and localized. The first recorded outbreak of EVD took place in Zaire (now the Democratic Republic of the Congo) in 1976, close to the Yambuku Catholic Mission Hospital located near the Ebola River Valley [7]. At the same time, a separate outbreak of EVD occurred near Maridi in West Equatoria Region in Sudan [7]. Prior to 2014, the largest recorded outbreak of Ebola (SEBOV in this case) took place in Uganda from October 2000 to January 2001, with 425 cases and 225 deaths [17, 18].

After the discovery of the virus, a large variety of organisms were screened as possible Ebola reservoirs. Bats both efficiently replicate the virus and survive infection, which made them standout as candidate reservoirs [4]. Despite this initial evidence, the first direct evidence that bats are reservoir hosts of *ebolavirus* was reported in a field study in 2005, almost 30 years after the discovery of the virus [19]. Immunoglobulin G (IgG) specific for *ebolavirus* was found in serum from bats of three different species of fruit bat (*Hypsignathus monstrosus, Epomps franqueti,* and *Myonycteris torquata*) and phylogenetic analysis showed that they were most likely close relatives of ZEBOV strains [19]. *Ebolavirus* antibodies have since been reported in numerous bat species from many locations, suggesting that infection (and survival) is frequent [4]. Retrospective analysis shows that wildlife deaths (non-human primates and antelope) tend to precede human infections, which this could have important surveillance implications in terms of preventing future outbreaks [20]. In addition, population declines in apes have also been chronologically linked to human Ebola outbreaks and a number of molecular studies have linked primate *ebolavirus* cases to human Ebola outbreaks [4].

Although two types of transmission (animal-to-human and human-to-human) have been observed, nosocomial transmission has played a key role in the spread of Ebola. Transmission from animals has taken place via the handling and butchering of infected animals, including bats, non-human primates and duikers (a small forest antelope) [21]. Healthcare workers are especially at risk for exposure to Ebola because they are more likely to come into contact with contaminated bodily fluids [22]. The traditional funeral and burial practices in West Africa involve washing the body by hand before burial and paying respect to the dead through physical contact which are both exceptionally high-risk activities with regard to the spread of Ebola [23]. The incubation period for Ebola can range from two to 21 days, but is usually one to two weeks [24]. There is no evidence that Ebola is contagious during the incubation period, while infected individuals are still asymptomatic [25]. The World Health Organization (WHO) will only declare an Ebola outbreak over once 42 days (two incubation periods) have passed with no new infections reported [26].

Interrupting Ebola transmission requires rapid identification of cases, contact tracing, and monitoring of people identified as high risk [22, 27]. Based on a retrospective study of the 1995 outbreak in Kikwit DRC, the greatest risk factor for secondary household transmission of Ebola is direct contact with someone who has clinically apparent illness [28]. This risk increases if there is contact with bodily fluids or the infected person is in the late stages of the disease. Direct contact was determined to be necessary, but not sufficient, for transmission [28]. To date, the only comprehensive analysis of viral excretion and environmental contamination from Ebola found viral particles are present in blood, breast milk, saliva, semen, feces, and tears [29]. A review of relevant literature by Thorsen et al. [30] found that the longest recorded persistence of EBOV in semen is 284 days. While viral RNA was isolated from this sample, it is not known if the viral particles were still infectious. Viable Ebola virus has been found in semen 82 days post-infection and may be present much longer than this [30]. Further complicating efforts to understand transmission was the recent report of sexual transmission confirmed by both contact tracing and genetic sequencing [31]. This case involved viral transmission from a male to a female 179 days after the onset of disease in the male patient [31].

The standard treatment for Ebola patients has not changed in the last 50 years and consists of symptomatic and supportive care [24]. Supportive care involves either oral or intravenous rehydration and electrolyte management; while symptomatic care involves the use of drugs to reduce vomiting and diarrhea, along with medication to treat fever and pain [32–34]. Patients with high malaria risk are also given anti-malarial medication and antibiotics to preemptively treat common infections that may hamper their ability to fight Ebola. Currently, drugs being developed to treat Ebola work by inhibiting viral replication either by targeting viral transcripts for degradation, blocking translation, or acutely neutralizing the virus [35]. Other treatments that are being studied include passive immunotherapy (blood transfusion from survivors) and mechanical filtering of patient blood [36, 37].

### Epidemiological dynamics of the 2014–2015 Ebola epidemic

**Fig. 1:**
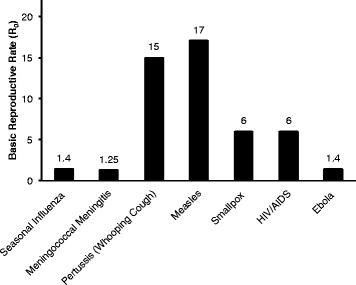
Average R_0_s for common epidemic diseases, including Ebola, at the height of the 2014 epidemic [43, 64–67]

Understanding epidemiological dynamics can be challenging during an outbreak when mortality rates are high and practical concerns, such as healthcare worker safety, need to be prioritized [38]. The main metric used to understand how fast a virus spread is R_0_, the basic reproduction number [39, 40]. For Liberia, estimates place R_0_ at 1.59, 1.36 and 1.83 according to three different studies [41] Pandey et al. 2014, [2]. In Guinea, R_0_ has been estimated at 1.5 and 1.71 [2, 41]. Three separate studies posit very different R_0_ values for Ebola in Sierra Leone. One estimates an R_0_ of 2.53, another estimates 2.02, and a third estimates 1.4 [2, 41, 42]. The third estimate was generated using a model based on clustered social interactions rather than assuming random mixing between individuals, and may therefore be more accurate [42]. According to the *United Nations* (UN) the overall R_0_ for the whole epidemic was approximately 1.4 in September (Fig. 1) and had fallen to below 1.0 in December 2014 [43].

### The 2014–2015 Ebola epidemic

All infections in the 2014–2015 West African ZEBOV epidemic can be traced back to an index case that was reported from an 18-month-old boy from the village of Meliandou, Guinea in December 2013 [43–45]. A retrospective investigation by Saéz et al. [46] posits that the index case was infected by contact with insectivorous bats. The first official medical alert was issued on January 24^th^, 2014 when the head of the Meliandou health post informed district health officials of five cases of severe and rapidly fatal diarrhea. A subsequent investigation by local health officials indicated that the symptoms appeared to match cholera (also endemic to this region), a conclusion later supported by bacteria found in patient samples [23]. On February 1^st^, 2014 Ebola reached Conakry, the Guinean capital, through an infected member of the index case’s extended family, who died 4 days later, but by that time had initiated multiple chains of transmission [23]. The Guinea Ministry of Health issued its first alert about the then unidentified disease on March 13^th^, 2014 and the regional office of the WHO opened an investigation the same day, suspecting Lassa fever (a hemorrhagic fever endemic to the region). The next day, the Pasteur Institute in France confirmed that the pathogen infecting patients was ZEBOV and on March 23^rd^, 2014 the WHO publicly announced the outbreak with 49 confirmed cases and 29 deaths [23].

From early June to mid-September, the epidemic grew exponentially in Guinea, Liberia, and Sierra Leone, with national case number doubling times of between 16 and 30 days [2]. Against this backdrop, the scaling up of the international response began on July 9^th^, 2014, when the United Nations Security Council issued a statement expressing its deep concern about the Ebola epidemic and implored the international community to provide prompt assistance to prevent the further spread of the virus [43]. On August 8^th^, 2014, the WHO declared the outbreak an international public health emergency [2]. Over a month later on September 18^th^, 2014, with 5,000 reported cases and almost 2,500 deaths, the UN Security Council held its first ever emergency meeting on a public health crisis [43].

The first recorded human-human transmission of EBOV outside of Africa occurred in Madrid, Spain. On September 30^th^, 2014, a nurse became sick after treating an Ebola patient who had been transferred to Spain from West Africa [47, 48]. She eventually made a full recovery and none of her contacts became infected. The next cases occurred in the United States and further ignited fears internationally of Ebola risk. Thomas Eric Duncan, a native of Liberia, flew from Liberia to Dallas, TX on September 19^th^, 2014 [49]. He became ill several days later and went to the emergency room of Texas Health Presbyterian Hospital on September 25^th^ where he was diagnosed with sinusitis and discharged with antibiotics [49]. He returned to the emergency room three days later in much worse condition and was admitted to the hospital [49]. Tests for EBOV came back positive on September 30^th^ and Duncan passed away on October 8^th^ [49, 50]. Subsequently, two nurses who had been involved in Duncan’s treatment became ill and tested positive for Ebola on October 12 and 15^th^, respectively [47, 50]. Both nurses made a full recovery and were released from the hospital [47].

On March 29^th^ 2016 the WHO declared the end of the Public Health Emergency of International Concern regarding Ebola in West Africa [51]. Liberia was initially declared Ebola-free on May 9^th^, 2015, however several more clusters of Ebola cases have occurred over the past year. A cluster of six cases was reported in June 2015, Liberia was eventually declared Ebola-free on September 3^rd^ 2015. Another cluster of three cases was reported in November 2015. Liberia was declared Ebola-free for the third time January 14^th^ 2015 and has not reported any new Ebola cases since that time [1]. Sierra Leone was first declared Ebola-free on November 7^th^, 2015. Two new cases were reported in January 2016; following these cases Seirra Leone was declared Ebola-free on March 7^th^ 2016 [1]. Guinea was declared Ebola-free on December 29^th^, 2015, but reported five new cases in late March 2016 [1].

### West Africa after Ebola

According to *Médecins Sans Frontières* (MSF; also known as Doctors Without Borders), no one knows the true number of deaths caused by the 2014–2015 Ebola epidemic [52]. The lack of basic healthcare means that overall morality rates have dramatically increased, in addition to deaths resulting from direct viral infections [52, 53]. For example, vaccination rates for common illnesses have also dropped—it is estimated as of March 2015 over a million more children will have not been vaccinated against measles than there were before the epidemic began [54]. The number of people who lack food security as a result of the 2014 Ebola epidemic is estimated to be in the hundreds of thousands and is expected to continue to rise [53]. Prior to the epidemic, healthcare in these countries was severely underfunded-- in 2012 the Liberian government spent $20 per person per year on healthcare, Sierra Leone $16 and Guinea $9. This is far below the minimum of $86 recommended by the World Health Organization to provide essential health services [53]. Including international aid, the total cost of the epidemic response is estimated at $4.3 billion USD so far [53].

Maternal health in West Africa has been dramatically affected by the Ebola epidemic. Pregnancy appears to make people more vulnerable to the effects of Ebola infection, particularly increasing their risk of hemorrhage [55]. All pregnancies of women infected with Ebola end in spontaneous miscarriage, stillbirth, or neonatal death within a few days [56]. There is evidence that the Ebola virus is able to cross from the placenta into both the amniotic fluid and fetus [56]. Besides the risks to the mother, the large amount of blood and bodily fluids present at deliveries present a huge risk of infection for healthcare workers [55]. A lot of the symptoms of pregnancy related complications overlap with EVD and this is further complicated by the poor condition of maternal health care in West Africa [56]. Many healthcare workers have refused to treat pregnant patients in countries with widespread Ebola infection until they have tested negative for EVD due to the risk of exposure, which poses a serious problem for women in need of invasive emergency procedures [56]. Refusal of treatment combined with fears about Ebola has meant that many people have stopped showing up for prenatal visits or assistance with delivery [55]. The United Nations Population Fund estimates that the Ebola epidemic will, either directly or indirectly, result in as many as 120,000 maternal deaths by the end of October 2015 [55].

Another area of concern is psychological care for Ebola survivors and family members of Ebola patients [57]. The epidemic has created many psychological stresses beyond fear of the disease itself, including declining economies, closed borders and markets, and widespread hunger [58]. Discrimination against families affected by Ebola and international stigma against countries with widespread Ebola infections also contribute to the development of mental health problems in affected communities [58]. There is a severe scarcity of mental health workers in West Africa making delivery of effective care even more difficult [58]. Currently, a shift in the global health community’s attitude toward mental health is resulting in more funding for mental health programs, and the WHO has started to address psychological care in its reports [57]. While these are encouraging signs, there is still a long way to go before Ebola survivors and families of Ebola victims receive adequate support.

### International engagement in public health

According to the WHO, Ebola in West Africa is an example of “an old virus in a new context”, which sums up some of the unique challenges faced during the Ebola response in West Africa [23]. From early June to mid-September 2015 the epidemic grew exponentially in Guinea, Liberia, and Sierra Leone, with national case number doubling times between 16 and 30 days [23]. Against this backdrop, the scaling up of the international response to the West African Ebola epidemic began on July 9^th^, 2014, when the United Nations Security Council issued a statement expressing its deep concern about the Ebola epidemic and implored the international community to provide prompt assistance to prevent the further spread of the virus [43].

As of April 2015, there were 176 organizations operating emergency programs in Guinea, Liberia, and Sierra Leone [59]. At this point, the total number of Ebola treatment unit beds exceeded the number of reported Ebola patients and there were enough burial teams in place to ensure safe and dignified burials for all deaths due to Ebola [59]. However, due to uneven distribution of these resources and the continued fear and suspicion of Ebola treatment hospitals and burial teams in local communities, many patients were still going without treatment or safe burials, resulting in new infections. According to the UN task force, the epidemic response needs to be tailored to adapt to the wide geographic spread of Ebola even as the outbreak diminishes [59].

According to the WHO, this outbreak demonstrated the severe lack of international capacity to respond to public health crises [23]. It has been estimated that more than 30,000 children were orphaned by this epidemic [23]. Access to routine healthcare has also been severely effected by the outbreak, resulting in increased mortality from common and chronic illnesses [59]. One year after the official declaration of the 2014 Ebola epidemic, MSF released a report critiquing the lack of international engagement with the epidemic response [52], specifically, MSF president Dr. Joanne Liu who pointed out that the lack of international political motivation to intervene in West Africa ended in July 2014 when the first case of Ebola was diagnosed *outside* of Africa [52]. It was only at this time that the outbreak could no longer be seen as a humanitarian crisis affecting a few poor countries in Africa, but instead began to be viewed as an international security threat to developed countries. This report by MSF also concluded that the interconnectedness of the modern world means that world leaders can no longer ignore health crises in distant countries [52].

As a result of this epidemic, several influential editorials have called for renewed attention to international public health issues. Bill Gates commented that the problem was less that the current system did not work well enough, but more that a system barely existed at all [60]. In his editorial published in the *New York Times*, Gates asserted that we must create a global warning and response system for outbreaks with a focus on building health systems within countries that can also be used for disease surveillance. He suggested creating a global warning and response system for outbreaks, increasing disease surveillance, and funding additional research into drugs, vaccines, and diagnostic tests, as well as creating a system for accelerating the approval of these interventions during a crisis [60]. Jeremy Farrar of the Wellcome Trust, and Seth Berkley of GAVI The Vaccine Alliance, argued that much more should have been done before this outbreak, in terms of vaccine studies and vaccine approval protocols [61]. Funding for research and development of drugs and vaccines for diseases likely to cause future epidemics, even though these are not the diseases that are the most lucrative for drug companies, may be a key component of preventing future outbreaks [61].

## Conclusions: what we have learned

The 2014 Ebola epidemic in West Africa highlighted major deficiencies in the ability of the international public health and scientific communities to respond to infectious disease emergencies. It also provided a stark reminder of the consequences of not investing in the development of healthcare infrastructure in developing countries. The current system of drug and vaccine development favors the development of drugs and vaccines for chronic diseases that primarily affect people in the developed world, rather than diseases likely to cause epidemics. According to Currie et al. [62], the development of a mechanism for international cooperation in vaccine development and licensing is an urgent priority.

The first step in preventing or minimizing future epidemics is to create an effective global monitoring system for newly emerging pathogens. This relies on improving healthcare infrastructure around the world, resulting in a network of healthcare professionals who could serve as an early warning system for disease outbreaks. It is important that knowledge from a variety of disciplines is employed to create a multifaceted approach to future outbreaks [62]. Another important facet of the global response to disease outbreaks is the rapid mobilization of personnel and resources. Thirdly, the Ebola outbreak has demonstrated the risk that international mobility and air travel poses to infection control, including the panic that can ensue when infected people move across international borders. The role of mobility and the importance of allocating resources to understand transmission and epidemiological risk has been underscored during the recent Zika virus outbreak, in part because of its previously unknown symptoms and transmission dynamics (reviewed in [63])

Ultimately the 2014 Ebola epidemic has shown that infection control measures can fail and that there is a significant risk from infectious disease worldwide. The risks posed by disease outbreaks are complicated by the lack of understanding of the basic biology, limited access to healthcare, poor infrastructure, and increased mobilization. Adequate scientific research and preparation, backed by careful policy implementation, are likely the key to limiting and responding effectively to future epidemics. Lessons learned from past outbreaks can be applied to prevent or minimize the impact of future outbreaks locally and globally.

## Abbreviations

BEBOV: *Bundibugyo ebolavirus*
CIEBOV: *Côte d’Ivoire ebolavirus*
EBOV: *Ebolavirus*
EVD: Ebola virus disease or Ebola
Kb: Kilobase
MSF: Médecins Sans Frontières
REBOV: *Reston ebolavirus*
*S*EBOV: *Sudan ebolavirus*
WHO: World Health Organization
ZEBOV: *Zaire ebolavirus*
